# Research landscape and emerging trends of diabetes-associated cognitive dysfunction: a bibliometric analysis

**DOI:** 10.3389/fnins.2023.1214301

**Published:** 2023-07-28

**Authors:** Siyi He, Qingchun Liang, Jie Zhu, Chen Wang, Xiafei Lin, Duozhi Wu, Wenqi Zhang, Guanwen Lin, Zhihua Wang

**Affiliations:** ^1^Department of Anesthesiology, Hainan General Hospital, Hainan Affiliated Hospital of Hainan Medical University, Haikou, Hainan, China; ^2^Department of Anesthesiology, The Third Affiliated Hospital, Southern Medical University, Guangzhou, Guangdong, China

**Keywords:** diabetes, cognitive dysfunction, bibliometric analysis, VOSviewer, CiteSpace

## Abstract

**Background:**

Diabetes-associated cognitive dysfunction (DACD) is a common and serious complication in diabetes and has a high impact on the lives of both individuals and society. Although a number of research has focused on DACD in the past two decades, there is no a study to systematically display the knowledge structure and development of the field. Thus, the present study aimed to show the landscape and identify the emerging trends of DACD research for assisting researchers or clinicians in grasping the knowledge domain faster and easier and focusing on the emerging trends in the field.

**Methods:**

We searched the Web of Science database for all DACD-related studies between 2000 and 2022. Bibliometric analysis was conducted using the VOSviewer, CiteSpace, Histcite, and R bibliometric package, revealing the most prominent research, countries, institutions, authors, journals, co-cited references, and keywords.

**Results:**

A total of 4,378 records were selected for analysis. We found that the volume of literature on DACD has increased over the years. In terms of the number of publications, the USA ranked first. The most productive institutions were the University of Washington and the University of Pittsburgh. Furthermore, Biessels GJ was the most productive author. *Journal of Alzheimers Disease*, *Diabetes Care*, and *Frontiers in Aging Neuroscience* had the most publications in this field. The keywords“dementia,” “alzheimers-disease,” “cognitive impairment” and “diabetes” are the main keywords. The burst keywords in recent years mainly included “signaling pathway” and “cognitive deficit.”

**Conclusion:**

This study systematically illustrated advances in DACD over the last 23 years. Current findings suggest that exploring potential mechanisms of DACD and the effect of anti-diabetes drugs on DACD are the hotspots in this field. Future research will also focus on the development of targeted drugs that act on the DACD signaling pathway.

## Introduction

Diabetes mellitus is increasing worldwide and is expected to affect approximately 642 million people by 2040 (Ogurtsova et al., 2017). Diabetes-associated cognitive dysfunction (DACD) is a complication caused by chronic hyperglycemia and microvascular diseases, which can lead to transient or permanent cognitive dysfunction (Kodl and Seaquist, 2008). Demographic trends for DACD very closely resemble those seen in diabetes mellitus (Biessels and Despa, 2018). Data from the large USA Veterans Registry showed that the prevalence of cognitive dysfunction in diabetes was 13.1% among those 65–74 years old and 24.2% among those 74 years and older (Biessels and Whitmer, 2020).

Type 1 diabetes mellitus (T1DM) can reduce cognitive abilities such as intelligence, processing speed, and mental flexibility (Kodl and Seaquist, 2008). While type 2 diabetes mellitus (T2DM) can specifically affect memory, processing speed, and executive function, which eventually results in cognitive dysfunction (McCrimmon et al., 2012). According to the severity, DACD can be classified into three approximate stages: asymptomatic preclinical stage, mild cognitive impairment (MCI), and dementia (Koekkoek et al., 2015). DACD has been found to significantly reduce an individual’s quality of life (Biessels and Despa, 2018), and increase their mortality (Biessels et al., 2020). Thus, DACD has received extensive attention from the medical community.

With further research, the number of studies on DACD is gradually increasing in recent years. Exploring the mechanism of DACD provides some clues, such as insulin resistance (IR), structural changes in brain tissue, altered cerebral blood flow, abnormal metabolism of brain cells, and impaired insulin signaling pathways (Stranahan et al., 2008; Geijselaers et al., 2015; Biessels and Despa, 2018). Additionally, daily care guideline on DACD has provided some advice on treatment and diagnosis methods (Koekkoek et al., 2015). However, there are still gaps in understanding the underlying mechanisms of DACD and improving therapeutic strategies (Biessels and Despa, 2018).

Bibliometric analysis is a powerful technique that can be applied to assess any particular research topic, predict emerging patterns, and reveal the research frontiers in a scientific field (Deng et al., 2022). Traditional literature reviews and systematic reviews cannot provide multiple perspectives in such an intuitive way as this approach (Yang et al., 2022). This method has been applied to analyze the hotspots of diabetes and its complications, assisting in the development of further research on disease prevention and treatment (Dehghanbanadaki et al., 2022; Zhang et al., 2022). Despite the extensive research conducted in the field of DACD in recent decades, there has been a lack of quantitative bibliometric analysis to assess the specific progress made in this area. Therefore, this study performed the co-authorship analysis of countries, institutions, authors, assessed the journal performances, and explored the emerging trends in the field of DACD using bibliometric analysis.

## Materials and methods

### Data source and search strategy

**Figure 1:**
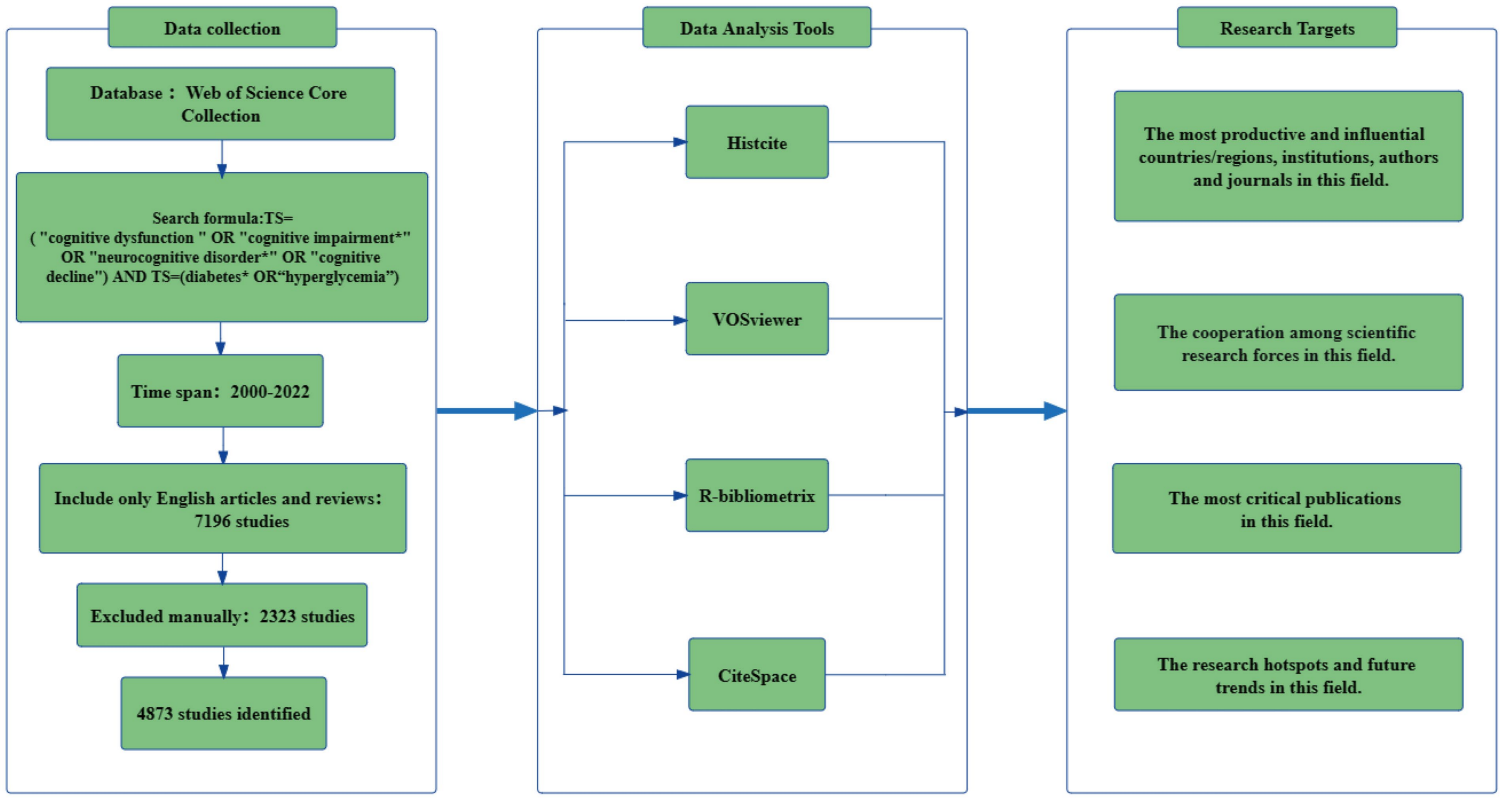
Flow chart of literature retrieval and analysis methods.

The Web of Science database was used to conduct a thorough search. To minimize potential bias from database updates, all searches were conducted on a single day. Two investigators (HSY and WC) conducted independently data retrieval on August 11, 2022. The search strategy was TS = (“cognitive dysfunction” OR “cognitive impairment*” OR “neurocognitive disorder*” OR “cognitive decline”) AND TS = (diabetes* OR “hyperglycemia”). The timespan of research included was from 2000 to 2022. We limited our data categories to “article” and “review” that were published in English. Meeting abstracts, proceedings papers, editorial material, book chapters and retracted publications were excluded from the literature. Additionally, we excluded some literature that was not related to our subject based on browsing the titles, abstracts, and full texts. Finally, we identified 2,323 possible inconsistent records, and only 4,873 publications were included. These included 3,821 articles and 1,052 reviews. The detailed process was shown in Figure 1.

### Softwares of social network maps

HistCite (Ke et al., 2020) was used to count the number of publications, the total local citation score (TLCS), and the total global citation score (TGCS) by each publication year, as well as screen the top countries, institutions, authors, and journals.

The software VOSViewer 1.6.15 (van Eck and Waltman, 2010) was employed to perform the authorship network and the co-occurrence network by using the counting method “full counting.” Different nodes in the graphic map represent items such as countries, authors, or keywords. The relevant quantity or number of items was reflected in the node size. Relationships of co-occurrence and collaboration were reflected by lines between nodes. Different clusters or matching years were reflected by the color of the node and line.

CiteSpace (6.1.R3) (Chen, 2004), a visual knowledge graph bibliometric tool based on the Java language, is commonly used to explore trends and future developments within particular topics. In this study, CiteSpace was mainly applied to analyze and display co-citation networks, timeline views, and citation bursts of reference and keywords. CiteSpace VI created a dual-map overlay of journals as well. According to the co-citation network, the results were displayed as clusters. The map of the visualization is made up of nodes and lines. Node size is dependent on the number of items, while links between nodes represent co-occurrences, collaborations, or citations. An indicator called centrality is used to gauge an element’s significance. When a purple ring surrounds an element with a centrality larger than 0.1, it shows that the element is reasonably significant (Chen, 2005). Modularity Q and mean contour are used to assess the main cluster analysis. The cluster structure is important enough to make the results believable when *Q* > 0.3 and mean profile >0.5 are present (Liu et al., 2021).

R-bibliometrix (Aria and Cuccurullo, 2017) was used to descriptively analyze the top research countries and journals. The h-index assesses the level of academic achievement of researchers, higher h-index indicates higher scholarly influence (Garfield et al., 2006). Meanwhile, derived from h-index, g-index can further measure scholars’ influence and academic achievements (Ali, 2021).

### Statistical analysis

Microsoft Office Excel 2021 served as the descriptive statistical analysis.

## Results

### Trends and annual publications

**Figure 2:**
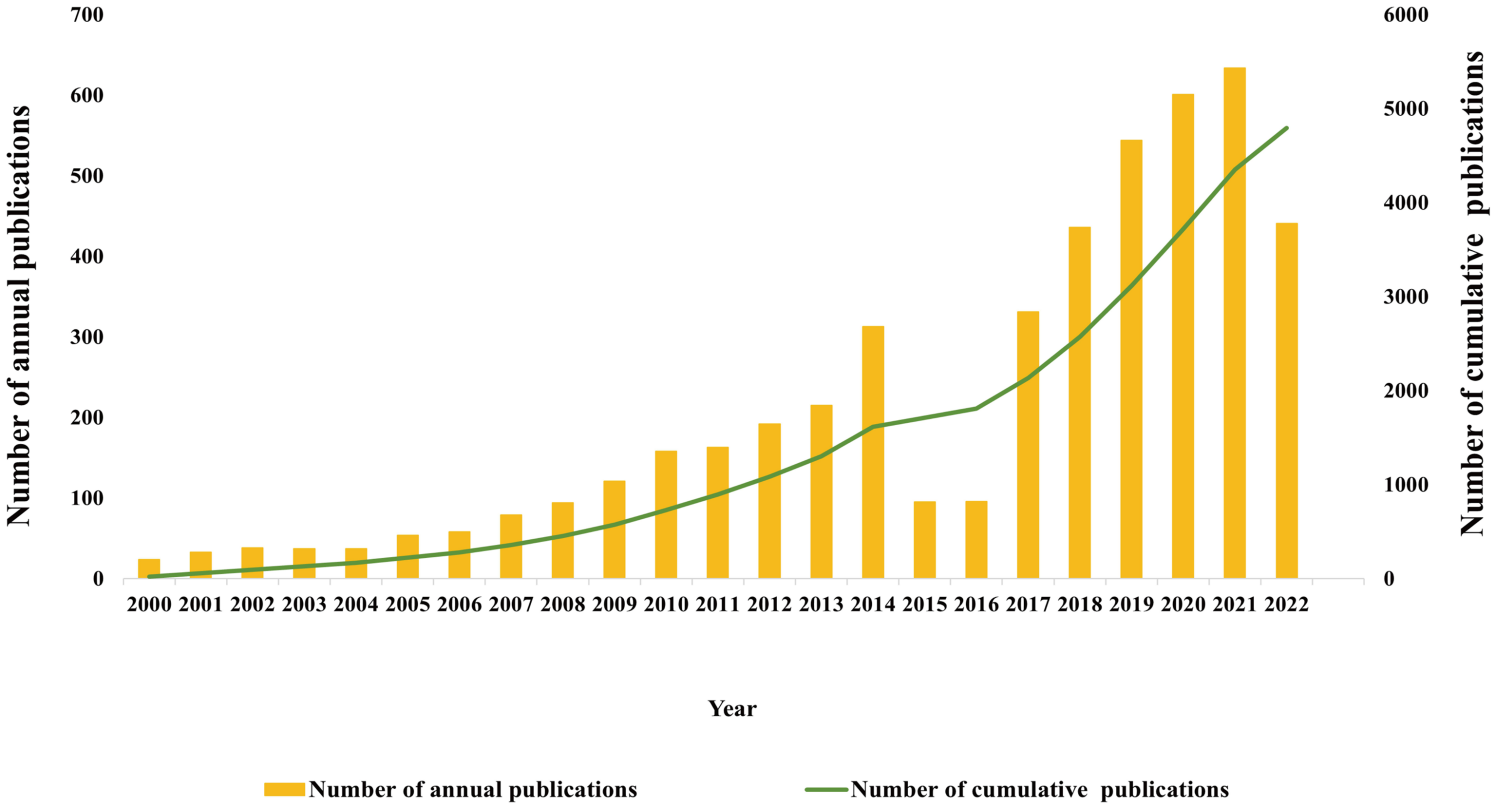
The total number of publications and the cumulative publications for research in DACD (2000–2022).

Scientific articles published in different periods reflect the popularity and development of the field. The number of annual publications and cumulative publications on DACD trended upward from 2000 to 2022 (Figure 2). In detail, more than 20 papers on DACD were published annually from 2000 to 2004. Thereafter, the number of publications grew steadily, going from 54 in 2005 to 313 in 2014. Although it declined in 2015 and 2016, it has grown rapidly in the recent 5 years and peaked in 2021 with 634. The number fell in 2022 due to incomplete trace time. Articles (3821) were almost four times more than reviews (1052) by document type. In order to better treat DACD, researchers have been working to identify the underlying mechanisms of DACD pathology and to find effective therapeutic targets. Therefore, in the last 23 years, there have been more original articles than review articles. In summary, the dynamic changes in publications suggest that research in this field has gradually matured over 23 years.

### Analysis of countries

**Figure 3:**
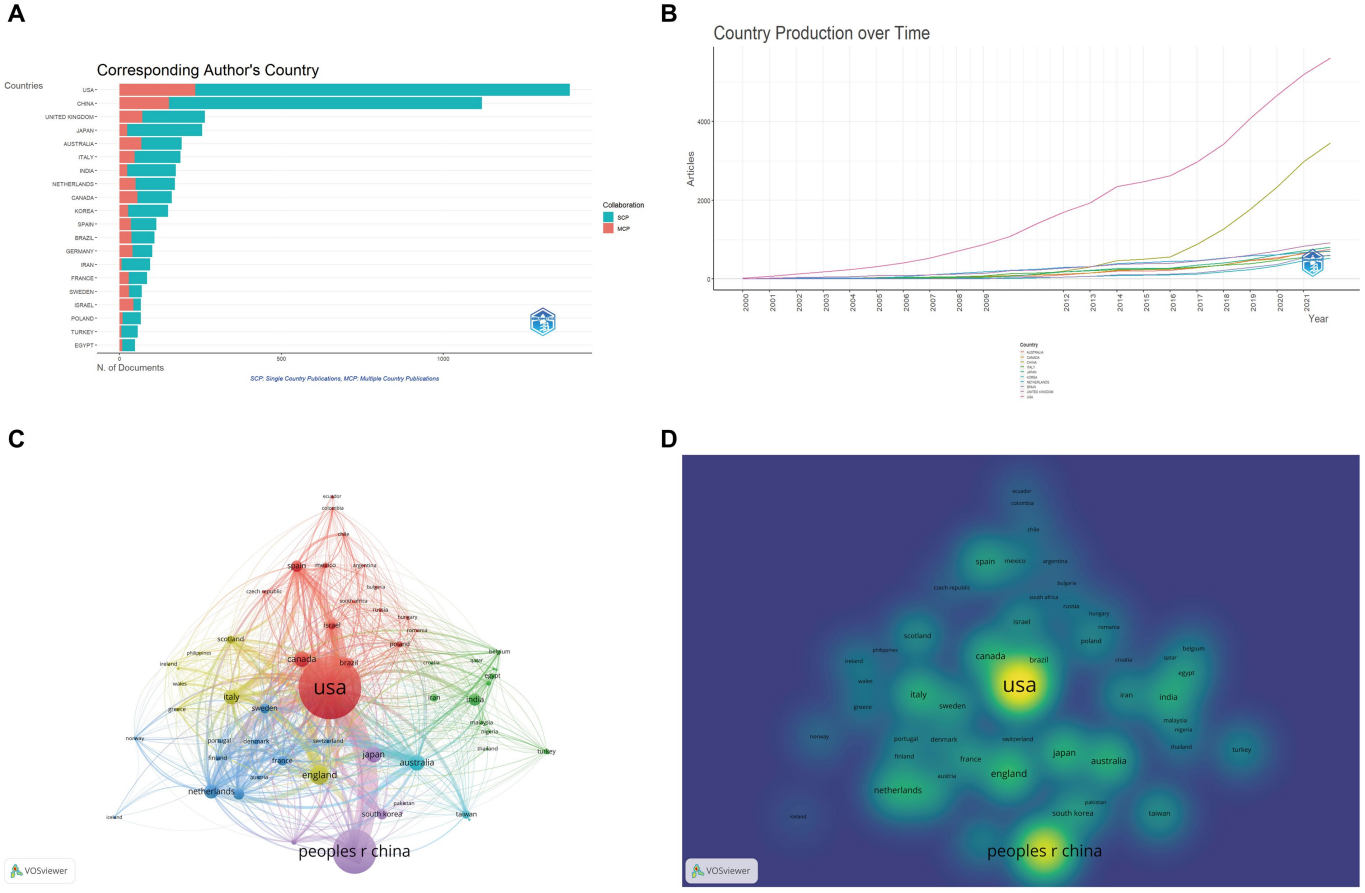
Diabetes-associated cognitive dysfunction (DACD) research contributions from different countries. **(A)** Top 20 corresponding author’s country. (MCP, multiple country publications; SCP, single country publications). **(B)** Top 10 countries’ production over time (2000–2022). **(C)** Network visualization of country collaboration. According to the map depicting countries’ cooperation, 61 countries had at least five publications. Each node represented a different country. Nodes are sized according to country publications, and the thickness of the links represents the strength of the relationship between them. **(D)** Density map of countries’ cooperation.

A total of 80 countries had contributed to the field of DACD. The USA had the most publications (1,222 articles), followed by China (997 articles) and the UK (234 articles). In addition, the USA also had the highest TGCS (84967), followed by the UK (19067) and China (17381) (Table 1). Figure 3B shows that both the USA and China had remarkable increases in production over time. Besides, the multiple-country publication (MCP) measures active and strong cooperation among different countries, the larger MCP means stronger cooperation between countries/regions (Sweileh, 2021). As illustrated in Figure 3A and Table 1, the top 3 countries demonstrating the strongest cooperation with other countries during the survey period were the USA (MCP = 193), China (MCP = 136), and the UK (MCP = 65). Countries owning 5 or more publications were used to form a network of collaboration in Figure 3C. The strongest collaboration was between the USA and China. As the number of items near the nodes increases, the weights of those items increase and the node color gets closer to yellow (van Eck and Waltman, 2010), so we can quickly identify active items in the field. According to Figure 3D, the USA and China were the most active countries in this field. Collectively, the above results demonstrate that the USA and China are the most influential countries in this field.

**Table 1:** Top 10 countries with the most published articles.

Country	Articles	TLCS^a^	TGCS^b^	SCP^c^	MCP^d^	MCP_Ratio
USA	1,222	11,806	84,967	1,029	193	0.158
China	997	2,338	17,381	861	136	0.136
UK	234	2,650	19,067	169	65	0.278
Japan	220	207	8,235	201	19	0.086
Australia	166	212	8,258	107	59	0.355
Italy	166	191	7,809	124	42	0.253
India	156	147	5,727	134	22	0.141
Netherlands	153	196	12,850	109	44	0.288
Canada	148	207	9,703	97	51	0.345
Korea	126	222	2,222	101	25	0.198

^a^TLCS, total location citation score. ^b^TGCS, total global citation score. ^c^SCP, single country publications. ^d^MCP, multiple country publications.

### Analysis of institutions

**Figure 4:**
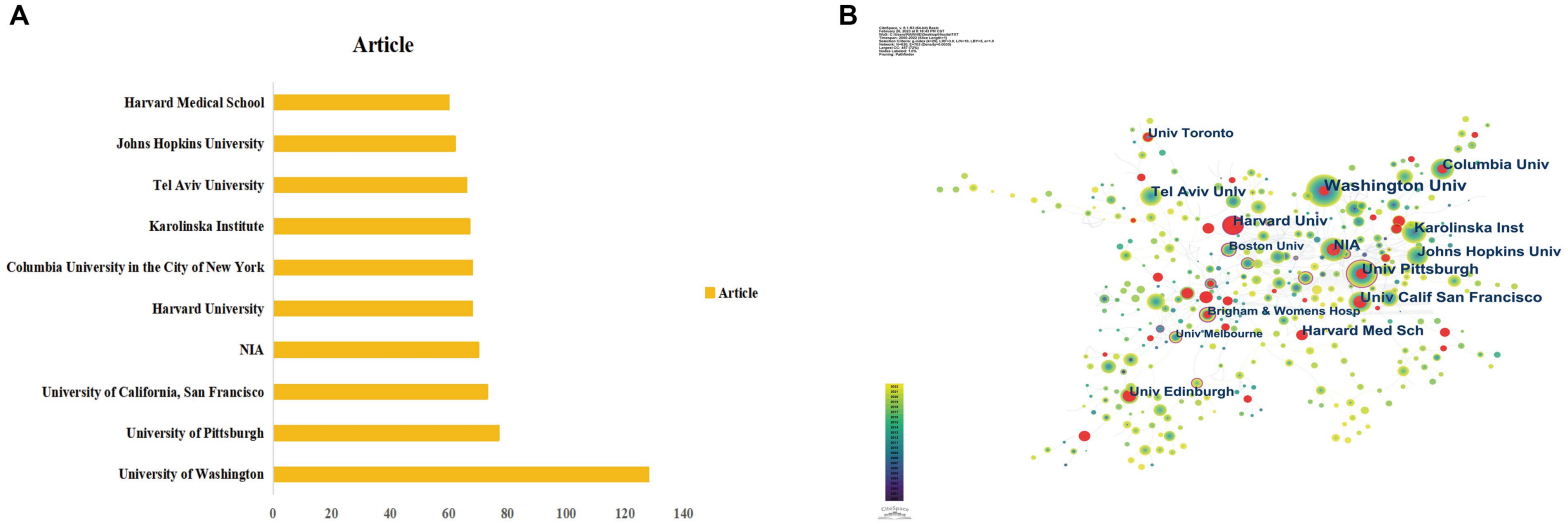
Analysis of institutions. **(A)** Top 10 productive institutions. **(B)** Network visualization of institution collaboration. Based on CiteSpace, the node’s size represents the number of publications from the institution, and the thickness of inter-institutional links indicates the strength of the institution’s relationship.

A total of 4,916 institutions have researched DACD. Figure 4A and Table 2 depicted the top 10 research institutions with the highest number of articles published. University of Washington (128 articles) ranked first by output, followed by the University of Pittsburgh (77 articles), and the University of California, San Francisco (73 articles). Additionally, the institutional co-authorship map was created using CiteSpace. Figure 4B demonstrates that institutions collaborate rather closely. Furthermore, the centrality of Duke University (0.24) and Boston University (0.23) was above 0.1, indicating that these institutions are critical hubs in promoting the development of this research area. Although Duke University was not the highest prolific institution, it had the highest centrality, implying that its articles have great influences. Finally, eight of the top ten most prolific institutions are from the USA, this implies that the USA is the dominant force in this field.

**Table 2:** Top 10 institutions referring to published articles or centrality.

Rank	Institution	Article	Country	Rank	Institution	Centrality	Country
1	University of Washington	128	USA	1	Duke University	0.24	USA
2	University of Pittsburgh	77	USA	2	Boston University	0.23	USA
3	University of California, San Francisco	73	USA	3	Brigham and Women’s Hospital	0.2	USA
4	NIA^a^	70	USA	4	University of Pittsburgh	0.14	USA
5	Harvard University	68	USA	5	University of Melbourne	0.14	Australia
6	Columbia University in the City of New York	68	USA	6	McGill University	0.13	Canada
7	Karolinska Institute	67	Sweden	7	Harvard University	0.13	USA
8	Tel Aviv University	66	Israel	8	University of Michigan	0.12	USA
9	Johns Hopkins University	62	USA	9	L’Institut national de la santé et de la recherche médicale	0.12	French
10	Harvard Medical School	60	USA	10	Radboud University Nijmegen	0.11	Netherlands

^a^NIA, National Institute on Aging.

### Analysis of authors and co-cited author

**Figure 5:**
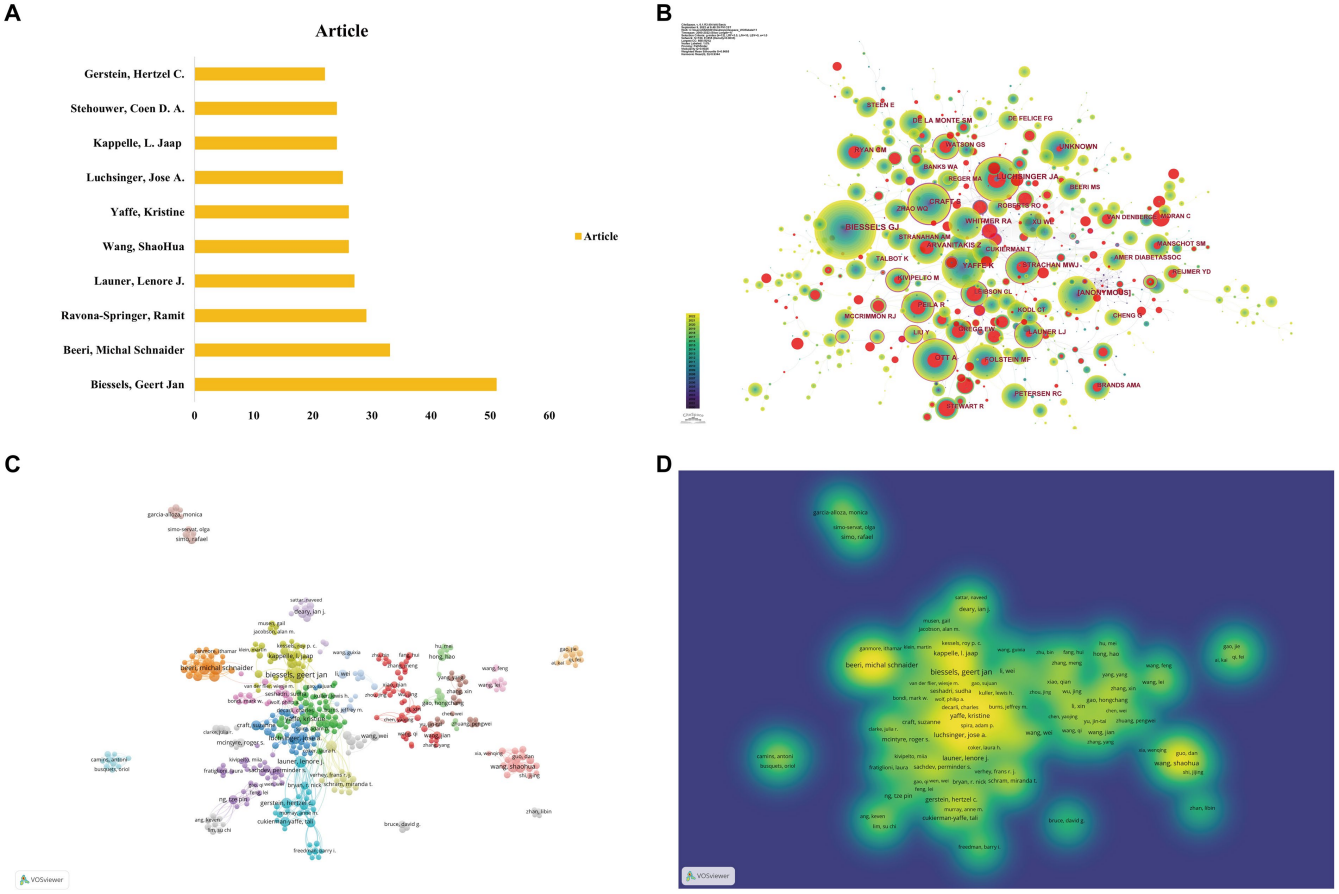
Analysis of authors and co-cited authors. **(A)** Top 10 authors in terms of the number of publications. **(B)** CiteSpace visualization of co-cited authors. **(C)** Network visualization of author collaboration. Approximately 1,000 authors with at least two publications were shown on the map of authors’ cooperation. The node’s size represents the the number of publications from the authors, and the thickness of inter-author links indicates the strength of the institution’s relationship. **(D)** Density map of authors’ cooperation on DACD.

To identify the most productive researchers in DACD over the last 23 years, all authors were ranked according to the number of publications. Table 3 and Figure 5A illustrated the top 10 productive authors in the field of DACD. Among them, Biessels GJ had the highest number of publications (51 articles), followed by Beeri MS (33 articles), and Ravona-Springer R (29 articles). Notably, although Yaffe K was not the author with the highest number of publications, she had the highest TGCS (7333) and H-index (123), indicating the importance of his research. The most frequently co-cited authors derived from the references are commonly used as a significant indicator to measure the author’s contribution to this field (Xu et al., 2022a). Visualization of the network of co-cited authors is shown in Figure 5B. The most co-cited author was also Biessels GJ (1,352 co-citations), followed by Luchsinger JA (744 co-citations) and Craft S (647 co-citations). Afterward, the collaboration network and density network of authors were constructed by VOSviewer, only authors with at least 2 publications were included in this analysis (Figures 5C,D). The analysis of the authors’ collaborative network divided the authors into more than 10 groups by different colors, represented by Biessels et al., Beeri et al., and Ravona-Springer et al. Overall, the above authors and their teams play an important role in this field and have strong academic impact.

**Table 3:** The top 10 productive authors referring to published articles.

Rank	Author	Article	Institution	Country	TLCS^a^	TGCS^b^	*H*-index
1	Biessels, Geert Jan	51	University Medical Centre Utrecht	Netherlands	1867	6,412	77
2	Beeri, Michal Schnaider	33	Chaim Sheba Medical Center Israel	Israel	202	832	30
3	Ravona-Springer, Ramit	29	Chaim Sheba Medical Center Israel	Israel	125	540	15
4	Launer, Lenore J.	27	NIA^c^	USA	589	2,477	107
5	Yaffe, Kristine	26	Univ Calif San Francisco	USA	1,175	7,333	123
6	Wang, ShaoHua	26	Southeast University Nanjing	China	92	307	33
7	Luchsinger, Jose A.	25	Columbia Univ	USA	630	2,185	62
8	Kappelle, L. Jaap	24	University Medical Center Utrecht	Netherlands	954	3,517	82
9	Stehouwer, Coen D. A.	24	Maastricht University	Netherlands	176	1,207	119
10	Gerstein, Hertzel C.	22	McMaster University Medical Centre	Canada	529	1950	4

^a^TLCS, total location citation score. ^b^TGCS, total global citation score. ^c^NIA, National Institute on Aging.

### Core journals

**Figure 6:**
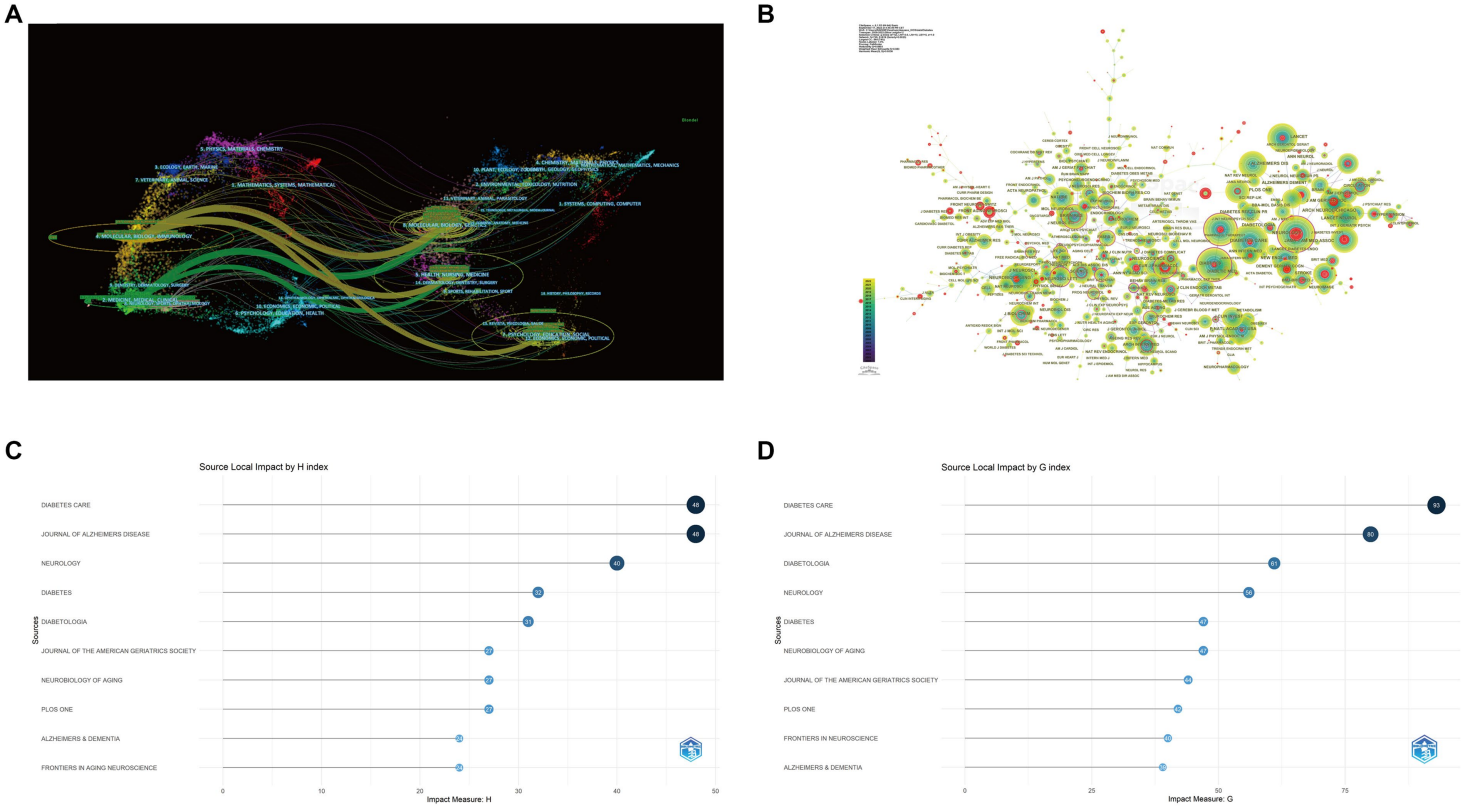
Analysis of journals. **(A)** A biplot overlay of journals on Diabetes-associated cognitive dysfunction. (Left side represents areas covered by citing journals, and the right side represents areas covered by cited journals). **(B)** CiteSpace visualization of co-cited journals. As a node in the network represents a journal, co-citations reflected by the size of the node. The centrality of a node with a purple ring around it signifies the importance of the journal. **(C)** TOP 10 journals in terms of H-index. **(D)** TOP 10 journals in terms of G-index.

To find the most popular publisher in DACD over the last 23 years, all journals were ranked according to the number of publications. Figure 6A shows a double map overlay of journals to illustrate the disciplinary distribution of journals based on DACD studies. Citation relationships are indicated by colored paths between the citing and cited journals. A two-color primary citation pathway is identified by the mapping, meaning that research published in journals in the field of molecular/biology/genetics and medicine/medical/clinical were primarily cited by research published in molecular/biology/immunology, medical/medical/clinical, health/nursing/medicine journals.

The journal with the most published articles was the *Journal of Alzheimers Disease* (177 articles), followed by *Diabetes Care* (85 articles) and *Frontiers in Aging Neuroscience* (64 articles). *Diabetes Care* has the highest IF (17.17) and TGCS (8119) among the top 10 most productive journals (Table 4). Figure 6B depicts a visual display of the co-cited journals, in the graph, co-cited journals are represented by circles, while connections between journals are denoted by lines. It revealed that *Diabetes Care* was the most co-cited journal (2,759 co-citations), followed by *Neurology* (2,681 co-citations) and *Diabetes* (2,411 co-citations). Furthermore, according to the *h*-index and *g*-index of the top 10 journals in Figures 6C,D, *Diabetes Care* also had the highest *h*-index (48) and *g*-index (93). Collectively, *Diabetes Care* is one of the most popular publishers and shows a high publishing potential in this field.

**Table 4:** The top 10 core journals referring to published articles.

Rank	Journal	Article	TLCS^a^	TGCS^b^	IF(2021)	JCR
1	Journal of Alzheimers Disease	177	1,209	7,073	4.16	Q2
2	Diabetes Care	85	1778	8,119	17.15	Q1
3	Frontiers in Aging Neuroscience	64	0	1,050	5.7	Q1
4	PLOS ONE	59	0	1707	3.75	Q2
5	Neurology	53	1,338	6,708	11.8	Q1
6	Diabetologia	49	1,077	4,525	10.46	Q1
7	Neurobiology of Aging	43	569	3,685	5.13	Q2
8	Diabetes	42	1,150	4,047	9.34	Q1
9	International Journal of Molecular Sciences	42	11	661	6.21	Q1
10	Frontiers in Neuroscience	42	0	1,346	5.15	Q2

^a^TLCS, total location citation score. ^b^TGCS, total global citation score.

### Co-cited reference analysis

**Figure 7:**
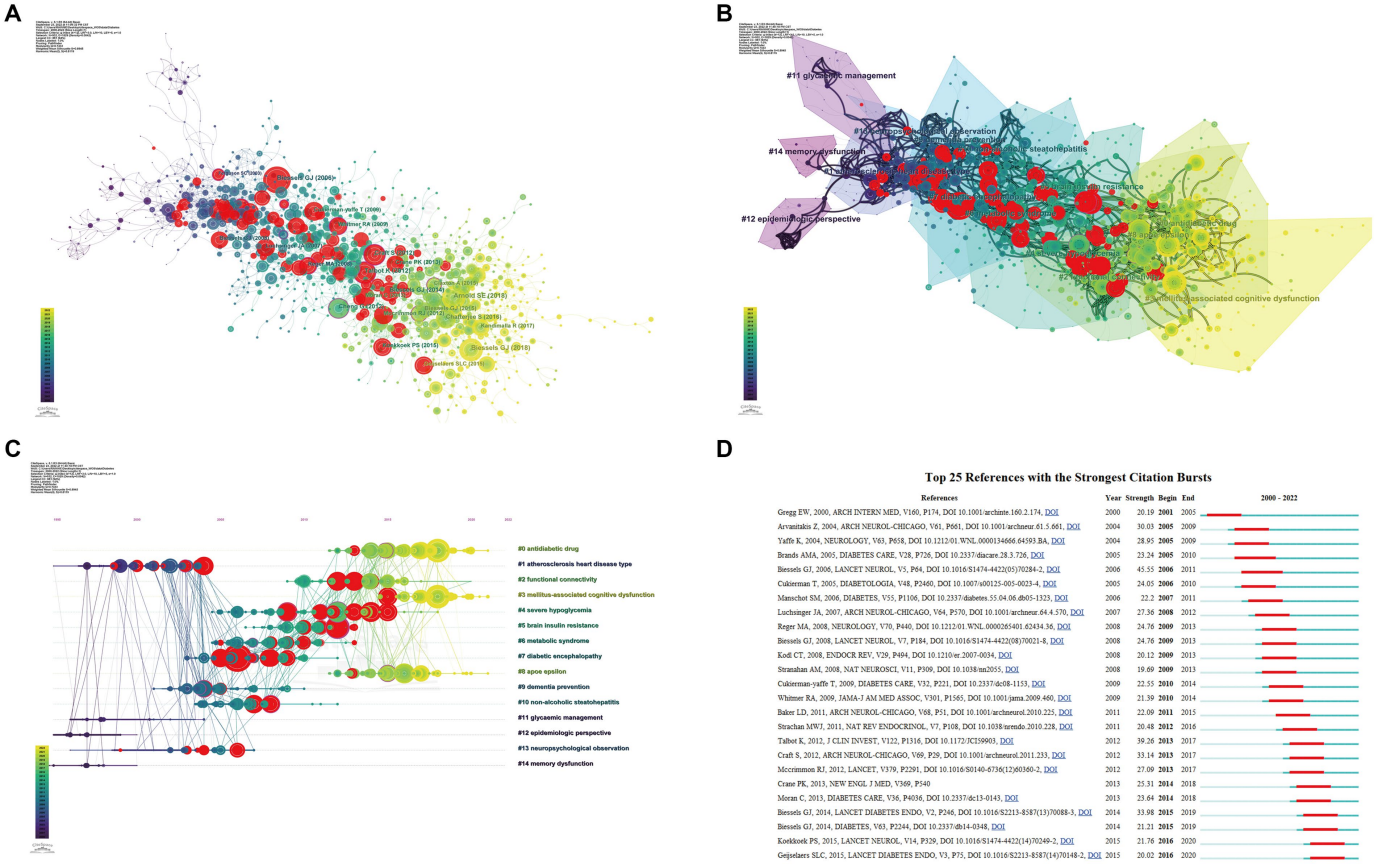
Analysis of co-cited references. **(A)** Visualization of co-cited references. Nodes represent co-cited references, with red circles representing citation bursts references. **(B)** Cluster analysis of co-cited references. A total of 15 clusters are found in the network graph. **(C)** Timeline graph of cluster analysis. **(D)** Top 25 references with the strongest citation bursts.

To find out the important articles in the field, co-cited references analysis was conducted using CiteSpace software. The visualized network of co-cited references contains 932 nodes and 1829 links (Figure 7A). Each cited article is represented by one node in the graph. The area of each node is related to the total co-citation counts of the associated article. In addition, the top 10 most co-cited references were listed in Table 5. Biessels’ article 2018 (Biessels and Despa, 2018) published in Nature had the highest co-citations (129), followed by Arnold et al. (2018) (112), and Biessels et al. (2006) (102). A total of 15 clusters with a modularity *Q* of 0.7433 and an average silhouette of 0.8119 were found by cluster analysis based on the log-likelihood ratio algorithm, indicating that the clustering results are credible. These clusters mainly included #0 “anti-diabetic drug,” #1 “atherosclerosis heart disease type,” #2 “functional connectivity,” #3 “diabetes-associated cognitive dysfunction,” #4 “severe hypoglycemia,” #5 “brain insulin resistance,” and #6 “metabolic syndrome” (Figure 7B). Besides, the research hotspots can be mirrored in the timeline map of the co-cited references (Figure 7C). Relatively, Cluster #3 “mellitus-associated cognitive dysfunction” and Cluster #0 “antidiabetic drug” were the hotspots in recent years. Moreover, the analysis of citation bursts can pinpoint articles that have drawn the attention of scholars in the same field, and screen articles that will have a significant impact on future research. Figure 7D shows the top 25 references with the strongest citation bursts. The first reference with the strongest citation burst appeared in 2001 (Gregg et al., 2000), and the latest references with the strongest citation bursts appeared in 2016 (Geijselaers et al., 2015; Koekkoek et al., 2015). Moreover, the (Biessels et al., 2006) had the strongest citation burst strength (45.55). The study aimed to show the association between diabetes and dementia as well as identify the risk factors and underlying mechanisms of DACD (Biessels et al., 2006).

**Table 5:** Top 10 co-cited references referring to co-citations.

Rank	Title	First author	Journal	Year	Co-Citations	Centrality	JCR	IF
1	Cognitive decline and dementia in diabetes mellitus: mechanisms and clinical implications.	Biessels GJ	Nature Reviews Endocrinology	2018	129	0.07	Q1	47.56
2	Brain insulin resistance in type 2 diabetes and Alzheimer disease: concepts and conundrums	Arnold SE	Nature Reviews Endocrinology	2018	112	0.07	Q1	44.71
3	Risk of dementia in diabetes mellitus: a systematic review	Biessels GJ	Lancet Neurology	2006	102	0.01	Q1	59.94
4	Demonstrated brain insulin resistance in Alzheimer’s disease patients is associated with IGF-1 resistance, IRS-1 dysregulation, and cognitive decline	Talbot K	Journal of Clinical Investigation	2012	91	0.14	Q1	19.46
5	intranasal insulin therapy for alzheimer disease and amnestic mild cognitive impairment:objective	Craft S	Archives of Neurology	2012	85	0.14	Q1	7.24
6	Dementia and cognitive decline type 2 diabetes and prediabetic stages: towards targeted interventions	Biessels GJ	Lancet Diabetes & Endocrinology	2014	83	0.06	Q1	44.87
7	Diabetes as a risk factor for dementia and mild cognitive impairment: a meta-analysis of longitudinal studies	Cheng G	Internal Medicine Journal	2012	81	0.14	Q3	2.3
8	Type 2 Diabetes as a Risk Factor for Dementia in Women Compared with Men: A Pooled Analysis of 2.3 Million People Comprising More Than 100,000 Cases of Dementia	Chatterjee S	Diabetes Care	2016	75	0.02	Q1	17.24
9	Hippocampal insulin resistance and cognitive dysfunction	Biessels GJ	Nature Reviews Endocrinology	2015	67	0.02	Q1	47.61
10	Cognitive function in patients with diabetes mellitus: guidance for daily care	Koekkoek PS	Lancet	2012	63	0.05	Q1	50.84

### Analysis of co-occurrence keywords’

**Figure 8:**
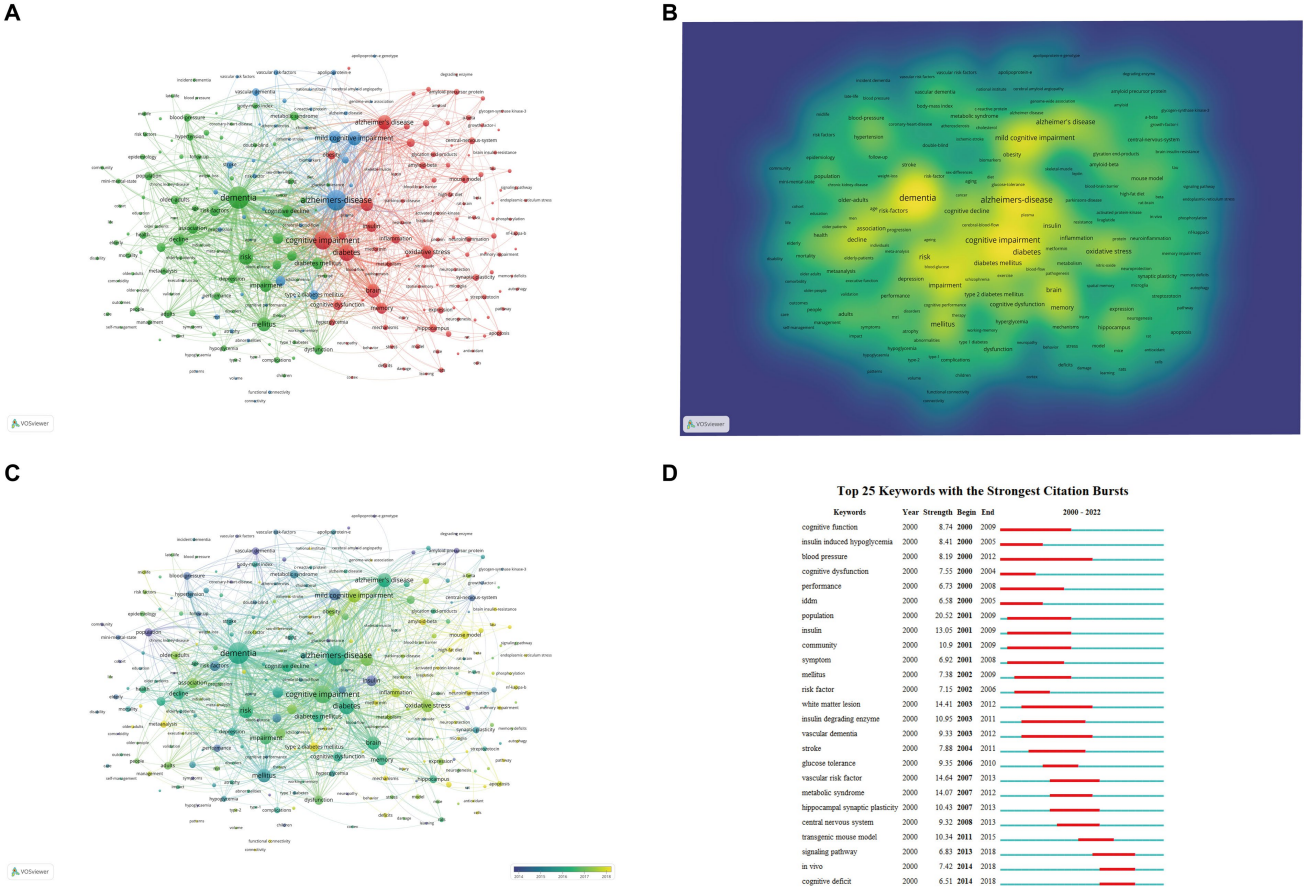
Analysis of keywords. **(A)** Co-occurrence and clustering of keywords in author keywords and keywords plus fields of publications related to DACD. **(B)** Density map of keywords co-occurrence. **(C)** Trends in keywords frequency over time. In the overlay visualization map, different colors were assigned to different keywords based on their average appearance time. In terms of time course, keywords in blue appeared relatively earlier than those in yellow. **(D)** CiteSpace visualization map of the Top 25 keywords with the strongest bursts.

Keywords reflect the theme of the article, through which the main points and research frontiers of a specific field can be analyzed. A total of 268 keywords with more than 30 occurrences were retrieved via the VOSviewer software. Then cluster analysis was carried out on the extracted keywords, and a total of three different color clusters were obtained, representing three research directions (Figure 8A). The most sizable cluster was cluster 1 (red), which contained 118 keywords related to “Alzheimer’s-disease,” “cognitive impairment,” “diabetes,” “Alzheimer’s disease,” “mild cognitive impairment,” “brain,” “oxidative stress,” and “insulin-resistance.” Cluster 2 (green) had 102 keywords. These keywords mostly covered “dementia,” “risk,” “impairment,” “diabetes mellitus,” “decline,” “cognitive decline,” “cognition,” “association,” and “risk factors.” A total of 48 keywords were presented in Cluster 3 (blue), such as “mellitus,” “cognitive dysfunction,” “type 2 diabetes mellitus,” “dysfunction,” “cognitive function,” “glucose,” “performance,” and “glycemic control.” The mechanism of DACD was predominantly reflected in Cluster 1. The keywords in Cluster 2 primarily reflected the disease-related risk factors of DACD. Most of the keywords in Cluster 3 referred to the effect of glycemic management on DACD.

According to Figure 8B and Table 6, “dementia,” “alzheimer’s disease,” “cognitive impairment,” and “diabetes” were the most frequently occurring keywords in the obtained literature. Furthermore, the average year of publication is used to determine the color of all keywords. “Type 2 diabetes mellitus,” “neuroinflammation,” “metformin,” “mitochondria,” “mechanisms,” “microglia,” and “amyloid-beta” were the most recent terms (Figure 8C). The top 25 strongest citation bursts keywords were extracted through keyword burst analysis, with the blue and red lines together forming the timeline (Figure 8D). The burst keywords that mainly focused on the mechanism (insulin-induced hypoglycemia, insulin, insulin degradation enzyme), and the change in the brain due to DACD (white matter lesions, central nervous system, hippocampal synaptic plasticity) began to explode in the beginning. Then, the recent burst keywords were “signaling pathway,” “*in vivo*,” and “cognitive deficit,” indicating that these research topics have received considerable attention recently and may become new research focuses in the years to come.

**Table 6:** Top 20 keywords referring to occurrence frequency.

Rank	Keyword	Occurrence frequency	Rank	Keyword	Occurrence frequency
1	Dementia	1,478	11	insulin-resistance	537
2	Alzheimer’s-disease	1,282	12	impairment	524
3	Cognitive impairment	1,045	13	diabetes-mellitus	516
4	Diabetes	832	14	diabetes mellitus	476
5	Risk	751	15	decline	469
6	Alzheimer’s disease	669	16	cognitive decline	459
7	Mild cognitive impairment	659	17	memory	425
8	Mellitus	639	18	cognition	413
9	Brain	612	19	insulin	408
10	Oxidative stress	567	20	association	385

## Discussion

This study is the first bibliometric analysis of global research on DACD. It can present the general trends in the field to researchers systematically and visually. We searched a total of 4,378 DACD-related articles and reviews published over the past 23 years. Although the number of publications has fluctuated over the decades, there has been an overall trend of growth in recent years. Our data revealed a burst of research activity after 2017. Biessels and Reagan (2015) pointed out that hippocampal insulin resistance maybe a potential mechanism of cognitive dysfunction in T2DM. Furthermore, Chatterjee’s finding further supported the role of diabetes in the etiology of dementia (Chatterjee et al., 2016). These findings provided new insights into DACD and have garnered the interest of researchers in the field, resulting in a surge of published papers.

The number of publications is considered to be a significant indicator to appraise the strength of national research. By this bibliometric analysis, the USA was the country with the largest number of publications, followed by China. This indicates that they have produced more in-depth studies in this field. The promotion of research in a certain field can be attributed to two important factors: governmental expenditure on healthcare and inter regional cooperation (Li et al., 2022). The USA spends $10,202 *per capita* on healthcare, outpacing most countries (Martin et al., 2022). Then, the USA has been a major contributor to DACD research, as can be seen from the fact that the majority of collaborations in this field are concentrated in it. The reasons for this include the presence of leading research institutions and funding opportunities. Thus, the USA has had a significant impact on the field over the past few decades.

Identifying core authors in the field can help researchers find potential collaborators. Our results on the analysis of authors showed that Biessels, GJ was the most productive and the most co-cited author, indicating that he is a key researcher in this field. His team have been focusing on mechanisms (atherosclerosis, microvascular disease, glucose toxicity, insulin resistance, inflammation, etc.) and treatments of DACD, as well as providing guidelines for the daily care of patients with DACD (Kamal et al., 2000; Brands et al., 2005; Koekkoek et al., 2015; Biessels and Despa, 2018). Furthermore, they identified and described the progress of cognitive dysfunction and its various stages, while also utilized MRI to determine the underlying structural changes in the brain (Manschot et al., 2006; van den Berg et al., 2006; Biessels and Reijmer, 2014; Brundel et al., 2014). Nine of the top 10 core authors came from developed countries, and only one from developing country China, American and European countries take the lead in this field, while research from China in this field has gained increasing attention in recent years. Studies by leading authors in the developed countries mainly focus on the link with the inflammatory, metabolic, vascular, hormonal factors between DACD, contributing to the early knowledge base relating to DACD. The study by Wang Shaohua, the only author from China, focused on prevention and treatment strategies for DACD, has explored the association between serum uric acid levels (Huang et al., 2019), lipoprotein-associated phospholipase A2 (Cai et al., 2017), serum IGF-1/IGFBP-3 molar ratio decreased (Huang et al., 2015) and cognitive functions in T2DM patients. Overall, the study on DACD showed close collaboration among the authors. Besides, most of the scholars engaged in DACD research were from different countries, and the cooperation was mostly confined to the research team. Therefore, the a fore-mentioned team members will produce more insightful articles, strengthening collaboration with these elite groups and across nations, leading to more notable advancements in DACD.

Identification of core journals can provide researchers with a wealth of reliable reference information and help them to screen the most suitable target journals (Zhuang et al., 2014). *Diabetes Care* may be the potential core journal in this field as the highest IF, TGCS, and co-citations. The journal *Diabetes Care* aims to improve the quality of patient care by catering to the needs of all healthcare professionals involved in the treatment of diabetes. Researchers in this field can prioritize this journal when they are searching for relevant references or submitting manuscripts.

The analysis of co-cited references provides insight into the core themes and key findings of current research (Wang et al., 2021). Most of the top 10 co-cited references concentrated on the pathophysiological mechanisms, treatment, and daily management of DACD. According to citation analysis, the most frequently co-cited reference is the publication by Biessels GJ in *Nature Reviews Endocrinology* in 2018 (Biessels and Despa, 2018). This article reviewed previous research in three areas: risk factors, brain imaging, and neuropathology. Several key clues to the underlying mechanisms of DACD and the future research focus were provided. Identifying the risk factors that affect the brain and contribute to the development of DACD through the observation of experimental models is a way to provide targeted treatment and prevention strategies for people with DACD. Hence, it had the highest cited and represented a high-level recognition of his research by other scholars. Another article with 112 citations was published in 2018 by Arnold SE. The team conducted a thorough review of experimental data and key observations on insulin signaling in the brain, emphasizing its effects on both neurons and glia. They proposed that both T2DM and Alzheimer’s disease (AD) are linked to cerebral insulin resistance and brain dysfunction (Arnold et al., 2018).

The combination of keyword cluster analysis and co-cited reference cluster can find hot topics (Xu et al., 2022b). Our results showed that signaling pathways were a hot spot and frontier area in this field. Recent popular pathways include the insulin–insulin receptor substrate (IRS) –Akt pathway, the phosphoinositide 3-kinase (PI3K) –Akt pathway, and the mitogen-activated extracellular signal-regulated kinase (MEK) –extracellular signal-regulated kinase (ERK) pathway (Arnold et al., 2018). Faulty activation of these signal pathways cascades results in impaired microvascular and mitochondrial function, and enhanced advanced glycation end products (AGE) and inflammation levels, which exacerbate oxidative stress (Biessels and Reagan, 2015; Grillo et al., 2015; Riederer et al., 2017; Arnold et al., 2018). These processesare usually associated with neurotoxicity, neurodegeneration, and cognitive deficits (Riederer et al., 2017). Moreover, brain insulin resistance has been verified to be an important pathway related to neurodegeneration and dysfunction in AD (Biessels and Reagan, 2015). Insulin receptors are widespread distribution in the brain, so the impairment of the insulin signaling pathway would influence development and function of major cell types of the brain (such as neurons, astrocytes, microglia, etc.) (Biessels and Reagan, 2015; Arnold et al., 2018). Neuron insulin resistance-induced deficits in synaptic plasticity, receptor regulation, or synaptic transmission, contributed to impaired regulation of metabolism or cognition dysfunction (Biessels and Reagan, 2015; Arnold et al., 2018). Similarly, astrocytic mitochondrial dysfunction, insulin resistance, and metabolic dysfunction may be also involved in the DACD pathology processes (Shen et al., 2023). In addition, a recent single-cell study found that activation of microglia in the hippocampus of db/db mouse promotes the expression of inflammatory factors and increase oxidative stress damage, which provides a novel strategy for screening DACD diagnostic biomarkers or potential therapeutic targets (Ma et al., 2022). Although studies above have provided some clues in this field, the pathogenesis of DACD is still not well understood. Future studies using single-cell sequencing analysis might be a trend to identify promising diagnostic biomarkers or potential therapeutic targets for DACD.

In recent years, more and more literature has focused on the impact of anti-diabetes drugs on DACD, which is becoming a new trend in the study of DACD treatment strategies. Earlier studies have demonstrated that insulin (Claxton et al., 2014), insulin sensitizer metformin (Koenig et al., 2017), dipeptidyl peptidase 4 (DPP4) inhibitors vildagliptin (Pipatpiboon et al., 2013), and peroxisome proliferator-activated receptor-γ (PPARγ) agonists rosiglitazone (Pathan et al., 2008) can improve cognitive dysfunction. Studies in rodent models show that insulin could improve cognitive performance by activating insulin receptor signaling in the hippocampus (Biessels and Reagan, 2015). Interestingly, compared to direct injection of insulin, intranasal insulin could directly supply insulin to brain target and penetrate the blood–brain barrier, thereby result in enhancing cognitive performance in mice (Chen et al., 2021). In adults with mild cognitive impairment, daily treatment with long-acting intranasal insulin could also mitigate cognition dysfunction (Claxton et al., 2014). Similarly, metformin, an insulin response enhancer, can improve cognitive dysfunction (Lin et al., 2018; Samaras et al., 2020) by modulating Akt/ glycogen synthase kinase 3 (GSK3) or cAMP response element binding (CREB) / brain-derived neurotrophic factor (BDNF) signaling pathway (Keshavarzi et al., 2019), and inhibiting cyclin-dependent kinase 5 (CDK5) hyper-activation and CDK5-dependent tau hyperactivation (Wang et al., 2020). Additionally, vildagliptin is a DPP4 inhibitor, which can enhance insulin sensitivity and prevent mitochondrial damage in the brain inhigh-fat diet-fed (HFD) rats (Pintana et al., 2013; Pipatpiboon et al., 2013). In elderly patients with type 2 diabetes, DPP4 inhibitor decreases the risk of cognitive dysfunction compared to sulfonylureas (Kim et al., 2019). In addition, the PPARγ agonists rosiglitazone reverses cognitive dysfunction by improving the peripheral insulin resistance in rats with high-fat diet (Pathan et al., 2008). The cognitive enhancement potential of anti-diabetic drugs has been evaluated in a number of preclinical and clinical studies (Chen et al., 2021). Nevertheless, these results are still needed to be verified by multi-center and large-sample clinical trials. In summary, from a pharmacotherapy perspective, identifying landmark anti-diabetes drugs that might improve the life quality and long-term outcomes of DACD patients are emerging trends in this field.

## Limitations

With the help of bibliometric analysis, this study systematically displays the research on DACD and captures the hotspots and emerging trends in this field. However, this study still has some shortcomings. Firstly, the study was limited to the Web of Science Core Collection database for literature screening, which may exclude a few relevant literature. This is due to current limitations in scientometric software, making combining multiple databases for analysis difficult. In the future, we will select more databases available for bibliometric analysis. Secondly, we only focused on the period from 2000 to 2022, thus we can not fully display the landscape of research on DACD. Therefore, we will try to select longer period for performing analysis to provide comprehensive information for this research field. Finally, only several tools such as VOSviewer, CiteSpace, Histcite, and R bibliometric package were used in this study, which may not fully interpret these data, we will try to perform the artificial neural networks in the following research using other tools.

## Conclusion

Bibliometric analysis shows that research on DACD is developing rapidly and has broad prospects. The most prolific country, institution, journal, and author are the USA, the University of Washington, *Diabetes Care*, and Biessels GJ, respectively. The reference with the most co-cited is written by Biessels, GJ in 2018. The research focuses are the underlying mechanism of DACD and the effect of anti-diabetes drugs on DACD. In addition, exploring new drugs targeting signal pathways for DACD is the emerging trend. In summary, this study systematically analyzes the literature on DACD, shows the landscape of research in the past decades, and provides direction for future research.

## Data availability statement

The original contributions presented in the study are included in the article/supplementary material, further inquiries can be directed to the corresponding authors.

## Author contributions

SH and QL designed the study plan and drafted the manuscript and retouched it. JZ and CW screened the literature. SH and XL performed the software analysis. ZW and DW revised the final version of manuscript and approved it. All authors contributed to the article and approved the submitted version.

## Funding

This study was supported by Hainan Provincial Natural Science Foundation of China (grant no. 2019RC365) and Guangzhou Key Laboratory of Neuropathic Pain Mechanism at Spinal Cord Level (202102100005).

## Conflict of interest

The authors declare that the research was conducted in the absence of any commercial or financial relationships that could be construed as a potential conflict of interest.

## Publisher’s note

All claims expressed in this article are solely those of the authors and do not necessarily represent those of their affiliated organizations, or those of the publisher, the editors and the reviewers. Any product that may be evaluated in this article, or claim that may be made by its manufacturer, is not guaranteed or endorsed by the publisher.
